# Effectiveness of group problem management plus in distressed Syrian refugees in Türkiye: a randomized controlled trial

**DOI:** 10.1017/S2045796024000453

**Published:** 2024-09-30

**Authors:** C. Acarturk, G. Kurt, Z. İlkkurşun, A. M. de Graaff, R. Bryant, P. Cuijpers, D. Fuhr, D. McDaid, A. L. Park, M. Sijbrandij, P. Ventevogel, E. Uygun

**Affiliations:** 1 Department of Psychology, Koc University, Istanbul, Türkiye; 2 School of Psychiatry and Mental Health, University of New South Wales, Sydney, NSW, Australia; 3 Department of Clinical, Neuro- and Developmental Psychology, WHO Collaborating Center for Research and Dissemination of Psychological Interventions, Amsterdam Public Health Research Institute, Vrije Universiteit Amsterdam, Amsterdam, The Netherlands; 4 School of Psychology, University of New South Wales, Sydney, NSW, Australia; 5 Babeș-Bolyai University, International Institute for Psychotherapy, Cluj-Napoca, Romania; 6 Department of Health Services Research and Policy, London School of Hygiene and Tropical Medicine, London, UK; 7 Department of Prevention and Evaluation, Leibniz Institute for Prevention Research and Epidemiology-BIPS, Bremen, Germany; 8 University of Bremen, Health Sciences, Bremen, Germany; 9 Care Policy and Evaluation Centre, Department of Health Policy, London School of Economics and Political Science, London, UK; 10 United Nations High Commissioner for Refugees, Public Health Section, Geneva, Switzerland; 11 Trauma and Disaster Mental Health, Istanbul Bilgi University, Istanbul, Türkiye

**Keywords:** anxiety, depression, psychological intervention, refugees, randomized controlled trial

## Abstract

**Aims:**

Despite high levels of psychological distress, mental health service use among Syrian refugees in urban settings is low. To address the mental healthcare gap, the World Health Organization developed group problem management plus (gPM+), a scalable psychological intervention delivered by non-specialist peer facilitators. The study aimed to evaluate the effectiveness of gPM+ in reducing symptoms of depression and anxiety among Syrian refugees in Istanbul, Türkiye.

**Methods:**

A randomized controlled trial was conducted among 368 distressed (Kessler Psychological Distress Scale, K10 > 15) adult Syrian refugees with impaired functioning (World Health Organization Disability Assessment Schedule, WHODAS 2.0 > 16). Participants were recruited between August 2019 and September 2020 through a non-governmental organization providing services to refugees. Participants were randomly allocated to gPM+ and enhanced care as usual (gPM+/E-CAU) (184 participants) or E-CAU only (184 participants). Primary outcomes were symptoms of depression and anxiety (Hopkins Symptom Checklist (HSCL-25)) at 3-month follow-up. Secondary outcomes were post-traumatic stress disorder (PTSD) symptoms (PTSD Checklist for Diagnostic and Statistical Manual of Mental Disorders-5; PCL-5), functional impairment (WHODAS 2.0), and self-identified problems (psychological outcome profiles).

**Results:**

Intent-to-treat analyses showed no significant effect of gPM+ on symptoms of anxiety, depression, PTSD and self-identified problems. Yet, there was a significant reduction in functional impairment in gPM+/E-CAU compared to E-CAU at 3-month follow-up (adjusted mean difference 1.66, 95 % CI 0.04, 3.27, *p* = 0.045, *d* = 0.19). Post-hoc subgroup analyses among participants with probable baseline depression or anxiety showed that there was a small but significant reduction in depression (adjusted mean difference −0.17, 95 % CI −0.32, −0.02, *p* = 0.028, *d* = 0.27) and anxiety (adjusted mean difference −0.21, 95 % CI −0.37, −0.05, *p* = 0.009, *d* = 0.30) symptoms comparing gPM+/E-CAU to E-CAU only at 1-week post assessment, but not at 3-month follow-up. There was a significant difference between conditions on functional impairment at 3-month follow-up, favouring gPM+/E-CAU condition (adjusted mean difference −1.98, 95 % CI −3.93, −0.02, *p* = 0.048, *d* = 0.26).

**Conclusion:**

In this study in an urban setting in Türkiye, gPM+ did not alleviate symptoms of depression and anxiety among Syrian refugees experiencing psychological distress and daily living difficulties. However, participants with higher distress at baseline seemed to benefit from gPM+, but treatment gains disappeared in the long term. Current findings highlight the potential benefit of tailored psychosocial interventions for highly distressed refugees in volatile low-resource settings.

## Introduction

The Syrian war has resulted in more than 6.6 million forcibly displaced Syrians and 360,000 civilian deaths to date (United Nations High Commissioner for Refugees, 2022). Syrian refugees mainly have fled to neighbouring countries such as Türkiye, Jordan, Lebanon and European countries. According to United Nations High Commissioner for Refugees, 3.5 million Syrians received temporary protection status in Türkiye by the end of 2022 (UNHCR, 2022). Currently, Türkiye hosts the highest number of refugees worldwide. Almost all Syrian refugees in Türkiye reside in urban settings (Presidency of Migration Management, 2023). Syrian refugees in urban environments must contend with several difficulties, such as lack of employment opportunities, bad working and housing conditions, insufficient food supply, discrimination by the host community and issues with access to services (Kurt *et al.*, 2022). This portends major challenges in responding to the needs of these refugees.

Refugees often have been exposed to potentially traumatic events such as the death of loved ones, torture, witnessing killings or destruction of houses before their flight. During their flight, they are also at heightened risk for various events including those related to safety and deprivation of basic needs. In the hosting country, various living difficulties such as discrimination, loss of social support networks and impoverishment may be ubiquitous (Silove *et al.*, 2017). Owing to these conflict and displacement-related stressors, refugees are at elevated risk for common mental disorders, including depression, anxiety and post-traumatic stress disorder (PTSD) (Acarturk *et al.*, 2021). A meta-analysis reported prevalence for depression, PTSD and anxiety of 32%, 31% and 11%, respectively among adult refugees and asylum seekers (Patanè *et al.*, 2022). Similarly, estimates for anxiety, depression and PTSD for Syrian refugees in Türkiye of 36.1%, 34.7% and 19.6%, respectively have been reported (Acarturk *et al.*, 2021). Moreover, the COVID-19 pandemic may have posed extra hardship on these populations. Given their existing vulnerabilities and poor living conditions, they may have been disproportionately affected by the pandemic. Indeed, COVID-19-related social and economic stressors significantly increased the risk of developing mental health problems among Syrian refugees (Kurt *et al.*, 2021).

Though several psychological treatments, such as trauma-focused cognitive behavioural therapy, interpersonal psychotherapy, eye movement desensitization and reprocessing, and behavioural activation, have been found effective in reducing psychological distress among refugees and asylum-seekers, including Syrian refugees in host countries (Turrini *et al*., 2019), access to and utilization of mental health services is very low among Syrian refugees in low-resource settings (Fuhr *et al*., 2020; Hendrickx *et al.*, 2020). To address the limited provision of mental health services in low- and middle-income countries, the World Health Organization (WHO) has developed brief, scalable transdiagnostic psychological interventions within its Mental Health Gap Action Programme (WHO, 2008). One intervention problem management plus (PM+) consists of four evidence-based strategies: (1) stress management (slow breathing), (2) problem solving, (3) behavioural activation and (4) accessing social support (Dawson *et al.*, 2015). This 5-session psychological intervention can be delivered by trained and supervised non-specialist peer facilitators, both in individual and group formats. Evidence shows that individual PM+ was effective in decreasing depression, anxiety and PTSD symptoms in Pakistan and Kenya (Bryant *et al.*, 2017; Rahman *et al.*, 2016). The group version of PM+ (gPM+) has been tested in previous trials, one with women living in a post-conflict setting in Pakistan (Rahman *et al.*, 2019) and one with adults who were affected by humanitarian disasters in Nepal (Jordans *et al.*, 2021), showing that gPM+ can significantly alleviate psychological distress by reducing symptoms of depression, anxiety and functional impairment. Furthermore, a recent trial (Bryant *et al.*, 2022) with Syrian refugees living in the Azraq refugee camp in Jordan was conducted within the current Syrian refugees mental health care systems (STRENGTHS) project (see Sijbrandij *et al.*, 2017). gPM+ was effective in reducing the symptoms of depression and personally identified problems (Bryant *et al.*, 2022). Further, a pilot randomized controlled trial conducted by our group in Istanbul showed that gPM+ is an acceptable, feasible and safe intervention, which warranting this larger trial to test its potential effectiveness (Acarturk *et al.*, 2022b).

The present study aimed to assess the effectiveness of a culturally adapted version of gPM+ in non-treatment-seeking mild to moderately distressed adult Syrian refugees living in Türkiye to decrease psychological distress, primarily depression and anxiety symptoms. Based on earlier studies examining the group version of PM+, our primary hypothesis was that gPM+ would be effective in reducing symptoms of depression and anxiety among refugees compared with enhanced care as usual (E-CAU). The secondary hypotheses were that gPM+ would reduce symptoms of PTSD symptoms, functional impairment and self-identified problems.

## Methods

### Study design

In this study, a two-arm, single-blind randomized controlled trial (RCT) comparing culturally adapted gPM+/E-CAU with E-CAU was conducted. The methods of the present study have been published in the protocol (Uygun *et al.*, 2020). The study was approved by the Ethics Committees of Istanbul Sehir University (Protocol ID: 12/2017), Koc University (Protocol ID: 2021.025.IRB3.006) and the Immigration Authority of the Republic of Türkiye. The trial protocol was pre-registered (ClinicalTrials.gov Identifier: NCT03960892, protocol version 4.2/201903). This study is reported according to the Consolidated Standards of Reporting Trials (CONSORT) guideline (Supplementary Material 1).

### Setting and study population

Participants were recruited between August 2019 and February 2020 through our implementing partner, the Refugees and Asylum Seekers Assistance and Solidarity Association (RASASA), a non-governmental organization (NGO). Since 2014, RASASA has been providing health, legal and social services to Syrian refugees in the Sultanbeyli suburb of Istanbul which hosts more than 19,000 Syrians. Adult Syrian refugees were informed about the study by the health and psychosocial staff of RASASA along with advertisements about the study on the website and social media accounts of the NGO. Syrian refugees who provided written consent to take part in the study were screened for eligibility to participate. Inclusion criteria were (a) being 18 years old or older, (b) having temporary protection status, (c) being an Arabic speaker, (d) reporting elevated levels of psychological stress (score >15 on the Kessler-10 Psychological Distress Scale (Kessler *et al.*, 2002)) and (e) reporting impaired psychosocial functioning (score >16 on the World Health Organization Disability Assessment Schedule (WHODAS 2.0) (Üstün *et al.*, 2010). Exclusion criteria consisted of having (a) an acute medical condition, (b) imminent risk of suicide (PM+ manual suicidality assessment), (c) indications of a severe mental disorder (e.g., psychotic disorders or substance use dependence; PM+ manual observation checklist) or (d) severe cognitive impairment (e.g. severe intellectual disability; PM+ manual observation checklist). Participants were excluded by the trained Arabic-speaking assessors based on the two sets of questions in PM+ manual (Appendix A of the Group PM+) on thoughts of suicide and impairments due to several mental, neurological and substance use disorders. The participants who were excluded on the basis of current psychosis, suicidal risk or dementia were referred to freely available mental health services at RASASA through the protection officer. The participants who were excluded for other reasons were referred to the available services at RASASA if they wanted to and were provided with the list of the available services.

### Randomization and blinding

Participants were randomly assigned either to gPM+/E-CAU or E-CAU only, on a 1:1 ratio by an independent researcher who was not involved in the rest of the study procedures. A computer-based random number generator (https://www.randomizer.org/) (Urbaniak and Plous, 2013) was used to produce unique random numbers to allocate participants into study arms, ensuring that each participant gets a distinct number and preventing any unintended repeated assignments. To achieve allocation concealment, a sequentially numbered list of group assignments was provided by the data manager to the trial coordinator in a password-protected online folder. The trial coordinator was not involved in the randomization process. Moreover, facilitators were not involved in outcome assessments, and outcome assessors were blind to the condition allocation of participants.

### Intervention

gPM+ is strongly protocolized and based on cognitive behavioural and problem-solving therapy (WHO, 2020). It consists of five weekly sessions, each of 2 hours delivered by (non-specialist) facilitators. In each session and using case examples, participants learn four strategies to help them cope with stress. Session 1 includes psychoeducation and stress management through a slow breathing exercise, session 2 focuses on learning a structured, step-by-step problem management strategy, session 3 is on behavioural activation and session 4 is on strengthening social support. Session 5 includes a review of the four strategies and relapse prevention. Participants are encouraged to participate in group discussions and contribute to the management of the problems presented in the case examples (WHO, 2020).

The gPM+ manual was adapted to ensure cultural appropriateness based on language, metaphors and contextual factors (Akhtar *et al.*, 2021). Before the commencement of trial, rapid qualitative assessments (free listing interviews, key informant interviews and focus group discussions) based on the Design, Implementation, Monitoring, and Evaluation model (Applied Mental Health Research Group, 2013) were conducted to understand important cultural and linguistic elements about Syrian culture. Adaptation recommendations based on rapid qualitative assessment were presented to key stakeholders including local and international healthcare providers, academics and policymakers (Akhtar *et al.*, 2021). The detailed cultural adaptation process was described elsewhere (Akhtar *et al.*, 2021).

For this trial, a non-specialist Syrian or other Arabic-speaking peer facilitator and co-facilitator (7 female, 3 male) delivered the sessions to groups of 8–10 participants. Following the cultural adaptation process and discussion with the Arabic-speaking research team, groups were separated by gender, and facilitators were matched by gender of the group. There was no request from any of the participants to be in the mixed-gender group. Non-specialist facilitators needed to have completed a minimum of 12 years of education. The majority of facilitators were university students at social sciences or other faculties such as engineering in Türkiye and had no prior experience in providing psychosocial support. Facilitators completed an 8-day training and weekly group supervision by two PM+ master trainers: one, a multilingual psychiatrist fluent in three languages (Arabic, English and Turkish) and the other, a PhD-level bilingual clinical psychologist fluent in two languages (English and Turkish). The supervision sessions were conducted in English, as all facilitators were fluent in English. The training of facilitators was conducted in English, but the trainer who spoke Arabic conducted some of the role plays in Arabic. The training of facilitators included classroom training covering basic helping skills, the gPM+ intervention protocol, group facilitation skills and suicide risk assessment and response. As the second component of the gPM+ training, facilitators completed two practice groups under supervision. The PM+ group sessions were conducted in RASASA during the weekend due to the childcare and work commitment of the participants. The same facilitators and co-facilitators facilitated the groups from the beginning. The facilitators and co-facilitators of the groups did not change throughout the intervention delivery; however, different combinations of facilitators facilitated different groups. Ten percent of all sessions were attended by a gPM+ supervisor to assess the treatment fidelity using a standardized checklist. As we iteratively evaluated the delivery of the intervention and provided supervision to the facilitators, no one demonstrated inadequate performance, thus excluded from the trial. An acceptable well delivery rate of 78% was achieved; therefore, no action was taken other than highlighting the importance of well delivery during the supervision sessions.

The facilitators were different from the assessors in the study. There were 15 female and 11 male Arabic speaking assessors who received a 2-day training that focused on basic helping skills, objectives of the project, design of the trial, the importance of standardization and blinding, potential safety issues and self-care. In case the assessors needed to refer anyone to specialized services, they liaised over the referral need with the protection officer at RASASA, who was responsible for connecting those participants with the relevant individuals in the organization if the participants wished to do so. There were no dropouts among the assessors.

### Enhanced care as usual (E-CAU)

Mental health service use is very low among Syrian refugees in Istanbul. Given that most of Syrian refugees in Istanbul do not know where to seek care and are concerned about the cost of care (Fuhr et al., 2019), we aimed to enhance care as usual condition through providing practical information on available free mental health services in Arabic. E-CAU was provided to all participants in both conditions. Participants were given leaflets to inform them about available free mental health services delivered in Arabic in primary healthcare centres, hospitals, and migrant healthcare centres and NGOs.

### Outcome measures

The primary outcomes were the symptom levels of depression and anxiety at 3-month follow-up assessed by the Hopkins Symptom Checklist (HSCL-25). The HSCL-25 contains 15 items for depression and 10 items for anxiety (Derogatis *et al.*, 1974). Items are rated on a 4-point scale (1 = not at all, 4 = extremely), with higher scores indicating more severe symptoms. HSCL-25 total score, depression and anxiety scores are calculated by taking the mean of all items, the 15 depression items and the 10 anxiety items, respectively (ranges: 1–4). The Arabic version of the HSCL-25 indicated a cut-off score of 2.00 and 2.10 for probable anxiety and depression, respectively (Mahfoud *et al.*, 2013). In the current study, the internal consistency was α = 0.87 for anxiety, α = 0.88 for depression and α = 0.92 for the total scale.

All other measures at post-intervention and the 3-month follow-up were secondary outcome measures. The severity of post-traumatic stress reactions was assessed by the PTSD Checklist for Diagnostic and Statistical Manual of Mental Disorders-5 (PCL-5) (α = 0.92) in which items are rated on a 5-point Likert scale (0 = not at all, 4 = extremely) (Ibrahim *et al.*, 2018). Self-identified problems were examined by the psychological outcomes profiles (PSYCHLOPS) (Ashworth *et al.*, 2004). Participants were asked to indicate two problems and evaluate the level of distress about those problems on a 6-point scale (with higher scores indicating greater severity), besides scoring their personal functioning and well-being on a 6-point scale (0–20). These items translated and back-translated into Arabic (α = 0.84). Functional impairment was examined by the WHO Disability Assessment Schedule 2.0 (WHODAS 2.0) (Üstün *et al.*, 2010), translated and back-translated into Arabic by the bilingual researchers in the team. The WHODAS questionnaire assesses the impairment in six domains with a 5-point Likert scale (1 = no difficulty, 5 = extreme difficulty) (α = 0.70). Higher scores indicate a higher level of functional impairment.

All the measures showed good to excellent reliability (above 0.70), pointing out that the items in the measures were measuring the same construct. The Arabic versions of outcome measures were administered by the trained Arabic-speaking assessors who were blind to the group allocation of the participants, at baseline, 1-week post-intervention (a week after the end of the intervention), and 3-month follow-up (primary endpoint, 3 months after the end of the intervention). All assessments were conducted in person at RASASA. The 12-month follow-up of this study will be reported elsewhere. A detailed overview of the study procedures is given elsewhere (Uygun *et al.*, 2020).

### Other measures

Lifetime trauma exposure at baseline was assessed with 27 items (range, 0–27) by a questionnaire adapted from the Harvard Trauma Questionnaire (Mollica *et al.*, 1992). Higher scores reflect exposure to a higher number of potentially traumatic events. Exposure to ongoing stressors was assessed by the Post-Migration Living Difficulties Checklist (PMLD), which assesses 17 stressors on a 5-point scale (Schick *et al.*, 2016; Silove *et al.*, 1997). Following the guidelines by WHO 2018, these two measures were translated and back-translated into Arabic. A modified version of the Client Service Receipt Inventory previously piloted, tailored to the Turkish context and translated into Arabic, was used by refugees to self-report health service utilization, as well as changes in informal care use and/or time in employment between baseline and 3-month follow-up (Acarturk *et al.*, 2022).

### Statistical analysis

We conducted power calculations based on the previous RCTs of PM+ (Bryant *et al.*, 2017; Rahman *et al.*, 2016). To detect a small to medium effect size with a power of 0.90 at an alpha level of 0.05, a minimum sample size of 133 participants per condition (266 in total) was required. Considering the potential 30% attrition rate at the 3-month follow-up assessment, we aimed to include a total number of 380 participants (190 in gPM+/E-CAU and 190 in E-CAU) in the definitive trial.

Intent-to-treat (ITT) analyses were conducted in R studio (R Core Team, n.d.) to test the effect of the intervention on primary (depression and anxiety [HSCL-25 total] and its subscales) and secondary outcomes (PTSD symptoms [PCL], self-identified problems [PSYCHLOPS], functional impairment [WHODAS 2.0]). Linear mixed models were performed to assess the treatment effect over time on average by coding time for both the 1-week post-assessment and the 3-month follow-up assessment as 1. To estimate the treatment effect for each assessment point separately, we created two dummy variables: one for the 1-week post-assessment and another for the 3-month follow-up assessment. We added the interaction term of condition by time as variable to the model, along with random intercept of the subject. The analyses were adjusted for the baseline values of each outcome measure. The regression coefficients of the interaction terms are the estimates of effect at each assessment point, representing the adjusted mean difference between the two conditions at each point.

The analyses were repeated with predefined covariates such as gender, age, education, baseline level of potentially traumatic experiences and baseline level of post-displacement stressors. To test the robustness of our findings, we performed two sets of sensitivity analyses, including participants who completed the 3-month follow-up assessment (completers only sample) and those who completed at least three gPM+ sessions (per protocol sample). We conducted a series of post-hoc analyses to examine whether treatment effects on the main outcome (depression and anxiety) differed between participants with vs. without baseline probable depression (mean score of >2.1 on depression subscale of HSCL-25) or anxiety (mean score of >2 on anxiety subscale of HSCL-25).

To determine whether the observed changes in the scores from baseline to 1-week post and 3-month follow-up assessments are reliable and clinically meaningful, we calculated reliable change index (RCI) scores for HSCL-25 total (Jacobson and Truax, 1991). Four categories of RCI scores were computed: recovered (substantial recovery in the symptoms), improved without recovery (reliable improvement in the symptoms but no recovery), deteriorated (reliable worsening of symptoms) and no change (no reliable and clinically significant worsening of symptoms). The recovery score was calculated by subtracting two standard deviations (*SD*) of the baseline HSCL-25 mean from each participant’s score at the post and follow-up assessment. Based on the following formula (Jacobson and Truax, 1991), the improved without recovery and deteriorated scores were calculated by using the baseline *SD* of HSCL-25 and baseline Cronbach’s alpha as the test-rest reliability coefficient. Participants whose RCI scores fall between ±1.96 are considered to show no reliable change in their scores. Those with RCI scores higher than 1.96 fall in the deteriorated category while RCI scores lower than −1.96 indicate reliable improvement in their symptoms at the respective assessment time. Chi-square significance tests were conducted to test whether the number of participants in each reliable change category was significantly different between the intervention and control condition at post-assessment times. Mean differences in the use of health services, as well as in days of productivity loss between baseline and 3-month follow-up between the two groups were analyzed and uncertainty in resource use cost distribution accounted for using bias-corrected and accelerated bootstrapping.

## Results

### Participants

**Figure 1.**
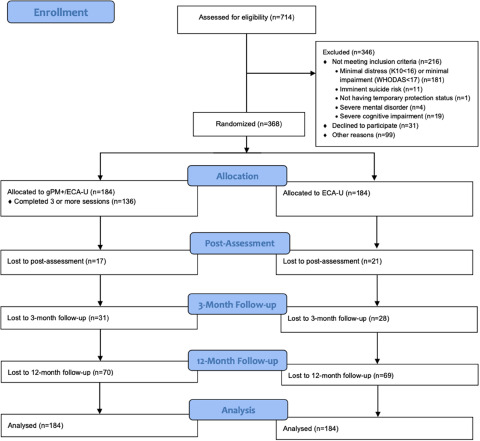
The CONSORT flowchart.

Participants were recruited to the study between August 2019 and September 2020. Follow-up assessments were completed in November 2021. 714 refugees were screened, of which 368 were found to be eligible and randomly assigned to gPM+/E-CAU (*n* = 184) and E-CAU (*n* = 184). Out of 714, 347 were excluded because of not meeting the inclusion criteria (*n* = 216), declining participation (*n* = 31) and other reasons (*n* = 99) (living in the same household as previously included participants). The CONSORT Flowchart is given in Fig. 1. Of the included participants, 256 were female (69.6%), with a mean age of 37.15 (SD = 11.21). The average time since leaving their home in Syria was 8 years (SD = 1.63, range = 3-12). The majority were married (303, 84.9%) and completed basic education (240, 67.6%). Baseline characteristics did not significantly differ between the two conditions. The most frequently reported potentially traumatic experiences were ‘being a civilian in a war zone’ (70.7%), ‘having been in danger during the flight (sea, boat, border)’ (54.6%) and ‘lack of food or water’ (53.3%) (Supplementary Material 2). In terms of moderately serious post-displacement stressors, participants most frequently experienced the following ones: ‘having not enough money to buy food, pay the rent or buy necessary clothes’ (84%), ‘difficulties obtaining financial assistance’ (81.5%) and ‘difficulties learning the local language’ (70.1%) (Supplementary Material 3).The sample characteristics are presented in Table 1.

**Table 1.** Baseline sample characteristics

	Total (*N* = 368)	gpm+/E-CAU (*N* = 184)	E-CAU (*N* = 184)
Female, *n* (%)	256 (69.6%)	133 (72.3%)	123 (66.8%)
Age, years, *M* (SD)	37.15 (11.21)	36.69 (10.83%)	37.6 (11.41)
Marital status, *n* (%)			
Single	18 (5%)	9 (5)	9 (5.1%)
Married	303 (84.9%)	153 (85.5%)	150 (84.3)
Separated	5 (1.4%)	3 (1.7%)	2 (1.1%)
Divorced	10 (2.8%)	5 (2.8%)	5 (2.8%)
Widowed	21 (5.9%)	9 (5%)	12 (6.7%)
Education, *n* (%)			
None	38 (10.7%)	11 (6.1%)	27 (15.4%)
Basic education	240 (67.6%)	130 (72.2%)	110 (62.9%)
Technical	27 (7.6%)	14 (7.8%)	13 (7.4%)
Secondary	33 (9.3%)	18 (10%)	15 (8.6%)
University and above	17 (4.8%)	7 (3.9%)	10 (5.7%)
Time since left Syria (in years), *M* (%)	8.01 (1.63)	8.12 (1.61)	7.90 (1.65)
Probable depression, *n* (%)	174 (47.8%)	83 (45.6%)	91 (50%)
Probable anxiety, *n* (%)	208 (57.1%)	103 (56.6%)	105 (57.7%)
Probable PTSD, *n* (%)	207 (56.3%)	98 (53.3%)	109 (59.2%)
Trauma, *M* (SD)	6.22 (3.98)	5.85 (3.95)	6.60 (3.99)
PMLD, *M* (SD)	8.16 (3.54)	8.21 (3.61)	8.11 (3.47)

The primary outcome measure (3-month follow-up) was completed for 309 participants (*n* = 153 in gPM+/E-CAU and *n* = 156 in E-CAU) with an attrition rate of only 16%. Except for gender (women were less likely to drop-out), participants who were lost to follow-up were not significantly different from those who were retained on any of the demographic or baseline characteristics (Table 2). Twelve percent of all sessions were observed for intervention fidelity and it was reported that 78% of the gPM+ components were delivered well.

**Table 2.** Baseline sample characteristics of retained at and lost to follow-up participants

	Retained at follow-up (*n* = 309)	Lost to follow-up (*n* = 59)	*t*/χ2	*P* value
Female, *n* (%)	224 (72.5%)	32 (54.2%)	7.80	0.005
Age, years, *M* (SD)	37.13 (11.18)	37.24 (10.91)	0.069	0.945
Marital status, *n* (%)			1.64	0.801
Single	14 (4.7%)	4 (7%)		
Married	255 (85%)	48 (84.2%)		
Separated	5 (1.7%)	0 (0%)		
Divorced	8 (2.7%)	2 (3.5%)		
Widowed	18 (6%)	3 (5.3%)		
Education, *n* (%)			2.34	0.673
None	31 (10.5%)	7 (11.9%)		
Basic education	197 (66.6%)	43 (72.9%)		
Technical	23 (7.8%)	4 (6.8%)		
Secondary	29 (9.8%)	4 (6.8%)		
University and above	16 (5.4%)	1 (1.7%)		
Time since left Syria (in years), *M* (%)	7.99 (1.64)	8.15 (1.58)	0.650	0.516
Probable depression, *n* (%)	143 (46.7%)	31 (53.4%)	0.881	0.348
Probable anxiety, *n* (%)	178 (58.2%)	30 (51.7%)	0.827	0.363
Probable PTSD, *n* (%)	168 (54.4%)	39 (66.1%)	2.77	0.096
Trauma, *M* (SD)	6.28 (4.11)	5.91 (3.27)	−0.643	0.520
PMLD, *M* (SD)	8.13 (3.45)	8.31 (4.09)	0.354	0.723

Effectiveness of gPM+ on primary and secondary outcomes.

Linear mixed models for the ITT sample showed that there was no significant mean difference between gPM+/E-CAU and E-CAU on any of the primary outcome measures at the 3-month follow-up assessment (adjusted mean difference −0.02, 95% CI −0.13, 0.09, *p* = 0.707, *d* = 0.03 for HSCL-25 total, adjusted mean difference −0.01, 95% CI −0.13, 0.11, *p* = 0.872, *d* = 0.02 for depression, and adjusted mean difference −0.04, 95% CI −0.18, 0.09, *p* = 0.533, *d* = 0.06 for anxiety).

Among secondary outcome measures, there was a significant mean difference between the two treatment conditions on the WHODAS (adjusted mean difference −1.61, 95% CI −3.12, −0.11, *p* = 0.035, *d* = 0.21), but not the PCL-5 or PSYCHLOPS In the gPM+/E-CAU condition, participants scored significantly lower on the WHODAS than those in the E-CAU at the 3-month follow-up assessment. No significant mean differences between gPM+/E-CAU and E-CAU were detected on any of the primary and secondary outcomes at 1-week post-assessment. All ITT results are presented in Table 3.

**Table 3.** Results from mixed-model analysis of primary and secondary outcomes

		Descriptive statistics		Mixed-model analysis			Covariate-adjusted mixed model analysis		
		Mean (SD)	Mean (SD)						
Outcome variable	Time point	gPM+/E-CAU (*n* = 184)	E-CAU (*n* = 184)	Difference in mean	*P* value	Effect size	Difference in mean	*P* value	Effect size
**HSCL−25 Total**	Baseline	2.12 (0.64)	2.16 (0.61)						
	Post	1.91 (0.57)	2.02 (0.64)	−0.08 (−0.19, 0.03)	0.170	0.13	−0.04 (−0.15, 0.07)	0.49	0.07
	3-month	2.08 (0.59)	2.13 (0.62)	−0.02 (−0.13, 0.09)	0.707	0.03	0.00 (−0.11, 0.11)	0.98	0.00
**HSCL−25 Depression**	Baseline	2.11 (0.66)	2.15 (0.64)						
	Post	1.88 (0.59)	1.99 (0.65)	−0.08 (−0.19, 0.04)	0.197	0.13	−0.05 (−0.16, 0.07)	0.45	0.08
	3-month	2.07 (0.62)	2.11 (0.66)	−0.01 (−0.13, 0.11)	0.872	0.02	0.02 (−0.09, 0.13)	0.74	0.03
**HSCL−25 Anxiety**	Baseline	2.12 (0.72)	2.18 (0.70)						
	Post	1.95 (0.64)	2.06 (0.71)	−0.08 (−0.21, 0.05)	0.217	0.12	−0.03 (−0.15, 0.10)	0.69	0.04
	3-month	2.09 (0.64)	2.17 (0.67)	−0.04 (−0.18, 0.09)	0.533	0.06	−0.02 (−0.15, 0.10)	0.71	0.03
**PCL**	Baseline	26.60 (17.72)	28.76 (17.76)						
	Post	23.14 (16.43)	22.81 (16.84)	1.46 (−1.79, 4.71)	0.379	0.09	1.68 (−1.55, 4.88)	0.31	0.10
	3-month	24.95 (15.87)	27.77 (17.86)	−1.20 (−4.55, 2.15)	0.482	0.07	−0.36 (−3.46, 2.71)	0.82	0.02
**WHO-DAS**	Baseline	26.67 (7.19)	25.77 (6.67)						
	Post	23.02 (6.92)	23.31 (7.85)	−0.81 (−2.27, 0.65)	0.277	0.11	−0.23 (−1.78, 1.36)	0.78	0.03
	3-month	23.34 (7.09)	24.75 (8.02)	−1.61 (−3.12, 0.11)	0.035*	0.21	−1.25 (−2.75, 0.28)	0.11	0.17
**PSYCHLOPS**	Baseline	14.06 (5.53)	14.70 (5.44)						
	Post	12.27 (5.49)	12.61 (5.86)	−0.15 (−1.38, 1.08)	0.815	0.03	0.21 (−1.12, 1.52)	0.76	0.04
	3-month	11.88 (6.13)	12.56 (6.46)	−0.44 (−1.72, 0.84)	0.499	0.07	−0.12 (−1.40, 1.15)	0.85	0.02

Planned covariate-adjusted analyses (gender, age, education, baseline trauma exposure and baseline post-displacement stressors) were similar to primary analyses without covariates, with the only difference for WHODAS 2.0. After adjusting for covariates, the observed significant effect on WHODAS 2.0 at the 3-month follow-up disappeared (Table 3 for the analyses with covariates). There were no significant differences in any element of health service utilization or productivity losses at 3-month follow-up (Supplementary Material 4). There remained very little use of all services in both groups.

The completers-only analysis focusing on participants who were retained at the 3-month follow up yielded similar results to the primary ITT analyses. The only significant mean difference between the two treatment arms at 3-month follow up remained on WHODAS 2.0 (adjusted mean difference 1.66, 95 % CI 0.04, 3.27, *p* = 0.045, *d* = 0.19) (Supplementary Material 5). Further, none of the mean differences on any of the primary and secondary outcomes was significantly different between gPM+/E-CAU and E-CAU at 1-week post or 3-month follow-up assessment when the analyses were repeated with the per protocol sample (Supplementary Material 6). Lastly, post-hoc subgroup analyses for participants with probable baseline depression or anxiety disorders (*N* = 233) showed that compared to those in E-CAU, participants in gPM+/E-CAU significantly scored lower on depression (adjusted mean difference −0.17, 95 % CI −0.32, −0.02, *p* = 0.028, *d* = 0.27) and anxiety (adjusted mean difference −0.21, 95 % CI −0.37, −0.05, *p* = 0.009, *d* = 0.30) at 1-week post-assessment. The mean difference between two conditions on WHODAS 2.0 at the 3-month assessment was significant (adjusted mean difference −1.98, 95 % CI −3.93, −0.02, *p* = 0.048, *d* = 0.26) (Supplementary Material 7).

The RCI analysis showed that at the 3-month follow-up, 42 participants (27.6%) in gPM+/E-CAU and 39 (25.3%) in E-CAU reliably improved in terms of their HSCL-25 scores, of which none recovered. The symptoms of 41 participants in gPM+/E-CAU (27%) and 34 in E-CAU (22.1%) were reliably higher at the 3-month follow-up compared to baseline. That is, their symptoms on HSCL-25 reliably and clinically significantly deteriorated at 3-month follow-up compared to the baseline assessment. Sixty-nine participants (45.4%) in gPM+/E-CAU and 81 (52.6%) in gPM+/E-CAU did not experience a reliable change in their scores at the 3-month follow-up. The number of participants in each category was not significantly different between the two treatment arms (χ2 (2, 306) = 1.71, *p* = .425).

Table 4 shows reliable change index at 1-week post and 3-month follow-up assessments.

**Table 4.** Reliable change index at post-assessment and 3-month follow-up for the HSCL-25 (completers only)

	1-week post-assessment		3-month follow-up	
RCI	gPM+/E-CAU	CAU	gPM+/E-CAU	CAU
Recovered, *n* (%)	0 (0%)	0 (0%)	0 (0%)	0 (0%)
Improved w/o recovery, *n* (%)^2^	55 (38.2%)	56 (40.6%)	42 (27.6%)	39 (25.3%)
Deteriorated, *n* (%)	25 (17.4%)	25 (18.1%)	41 (27%)	34 (22.1%)
No change, *n* (%)	64 (44.4%)	57 (41.3%)	69 (45.4%)	81 (52.6%)

## Discussion

This RCT tested the effectiveness of gPM+ to alleviate psychological distress among distressed Syrian refugees living in an urban setting in Türkiye. In contrast to our expectations, the results yielded that there was no significant difference in primary outcomes (depression and anxiety symptoms at the 3-month follow-up assessment) between the gPM+/E-CAU and E-CAU. gPM+ did not lead to significant reductions in any of the secondary outcome measures except for functional impairment. gPM+ was also not associated with any negative outcomes such as deterioration in the symptoms. However, we found a significant effect of gPM+ on reducing symptoms of depression and anxiety at the 1-week post-assessment for those highly distressed at the baseline.

Although our main findings are somewhat inconsistent with the previous trials on PM+, this trial highlighted the potential of gPM+ in reducing the psychological burden among those initially highly distressed. Previous trials found that both individual (Bryant *et al.*, 2017; Rahman *et al.*, 2016) and group versions of PM+ (Rahman *et al.*, 2019) effectively reduce depression and anxiety among people living in low-resource settings including conflict-affected areas. These findings were not obtained from refugee populations but a recent study among Syrian refugees living in a camp in Jordan reported similar benefits: gPM+ led to a significant decrease in depression symptoms and self-identified problems among participants in the intervention group (Bryant *et al.*, 2022). This is further supported by the findings of a recent trial testing individual PM+ among Syrian refugees in the Netherlands (de Graaff *et al.*, 2023). Taken together, previous studies showed effectiveness of both individual and group PM+ in reducing depression symptoms among high-risk populations, including refugees. On the other hand, similar to our findings, the previous gPM+ trials in conflict and disaster-prone settings and among Syrian refugees in Jordanian camps did not find effects on PTSD symptoms and anxiety symptoms (Bryant *et al.*, 2022; Jordans *et al.*, 2021). However, individual PM+ was consistently found to reduce PTSD symptoms (Bryant *et al.*, 2017; de Graaff *et al.*, 2023; Rahman *et al.*, 2016), which might signal the need for personalized care to reduce PTSD symptoms.

The results on the effectiveness of PM+ on functional impairment are, so far, equivocal. A study with women who experienced gender-based violence in Kenya found a small effect of individual PM+ on functional impairment (Bryant *et al.*, 2017). Similarly, two clinical trials in Pakistan showed a moderate effect of individual PM+ and group PM+ on reducing functional impairment in the conflict-affected setting (Rahman *et al.*, 2016, 2019). However, a recent study on gPM+ with Syrian refugees in Jordan (Bryant *et al.*, 2022) did not find an effect on functional impairment. These mixed findings point out the potential interplay of contextual factors and implementation processes in those trials. Contextual factors play a significant role in determining the implementation process of an intervention including the delivery, uptake and engagement (Le *et al.*, 2022). Socio-political, economic and cultural differences across those implementation settings might explain the divergent outcomes of the trials. Those findings, also, raise the question as to the working mechanism of PM+. So far, only one trial has investigated the working mechanism whereby PM+ leads to significant improvement in psychological distress. Jordans *et al.* (2021) showed that the high level of psychosocial skills acquisition explained the observed effect of gPM+ on reducing psychological distress. Despite potential utility, this finding did not provide information as to the acquisition of which strategy or strategies are the main drivers of the change. Which strategies work for refugees living in highly unstable contexts warrants further inquiry.

Our trial also showed that gPM+ was moderately effective in reducing the symptoms of depression and anxiety at 1-week post-assessment for Syrian refugees with probable depression or anxiety disorders at baseline. This finding was consistent with the results of a meta-analysis that compared low-intensity interventions with usual care (de Graaff *et al.*, 2023; Karyotaki *et al.*, 2018, 2021). It was reported that the participants with more severe depression symptoms (i.e., scoring above cut-off) at baseline assessment benefitted more from the interventions compared to the participants with lower depression symptoms (i.e., scoring below cut-off) at baseline. Similarly, the results of the current study show the potential of gPM+ to be effective for highly distressed refugees although PM+ is a non-specialized psychological intervention and situated at the third-level of the Interagency Standing Committee (IASC)’s framework for mental health and psychosocial support services (‘focused non-specialist supports’ level; IASC, 2008). With this finding, PM+ can be also offered as a treatment for those with higher symptom levels considering it was suggested that those with higher symptom levels may be more motivated to change (Conejo-Ceron et al., 2020). However, non-maintenance of treatment gains at the 3-month follow-up assessment signals the need for identifying strategies to maintain treatment gains in the longer-term. Booster sessions can help retaining treatment gains (Bryant, 2023).

There are several explanations why our main findings on depression and anxiety symptoms differ from those of earlier trials. First, Syrians have a temporary protection status in Türkiye, giving them access to basic services such as education and health. Although the temporary protection status does not preclude them from utilizing basic services, it leads to a constant sense of temporariness. Indeed, our analysis indicated that health service utilization in both groups remained very low. Uncertainty around legal status creates additional difficulties which are likely to be beyond individuals’ capacity to manage or actively work on (Li *et al.*, 2016; Nickerson *et al.*, 2019). As such, in this context of ongoing uncertainty, strategies focusing on problem management may not have yielded the intended outcomes in the current study. In such circumstances, psychosocial interventions targeting individuals’ emotion-focused coping capacity might better help deal with the feelings elicited by uncontrollable external conditions. Recently, self-help plus (SH+), a scalable psychosocial intervention focusing on fostering psychological flexibility, was found to be effective in preventing the onset of mental health disorders among Syrian refugees in Türkiye (Acarturk *et al.*, 2022). SH+ is also effective in mitigating psychological distress in low-resource settings (Tol *et al.*, 2020).

Further, it is evident that post-displacement stressors play a pivotal role in determining the course and outcome of psychological treatments (Schick *et al.*, 2018). Living in an ever-changing resettlement context might hinder individuals from obtaining significant gains from psychological treatments. Therefore, focusing on post-displacement stressors might contribute to the successful treatment of mental health problems. In a recent feasibility trial with Afghan refugees and asylum-seekers in Austria (Knefel *et al.*, 2022), an additional session was added to PM+ to tackle the burden caused by post-displacement stressors. In that session, participants were given the option to choose between anger regulation and increasing self-efficacy strategies. The anger regulation strategy focuses on the acquisition and improvement of coping skills for effective management and acceptance of intense anger related to post-displacement stressors. Increasing self-efficacy strategy, on the other hand, aims to help individuals to uncover their competencies and strengths to deal with those stressors. Results showed that this adapted version of PM+ significantly reduced the symptoms of anxiety and depression, and distress related to the stressors and improved the quality of life among Afghan refugees. Thus, such adaptation might be beneficial for those experiencing a high level of post-displacement difficulties. Integrated mental health services with components targeting the social determinants (e.g., housing aid and cash transfer) might be also effective in promoting and protecting mental health of refugees (Lund *et al.*, 2018).

This study has several strengths, including high retention of participants at follow-up assessments and the delivery of the intervention by non-specialist peer facilitators, which appeared to be feasible and safe as evident in the results for reliable change in the symptoms. There are several limitations to this study. First, the trial only included Syrians with temporary protection status. Thus, it is not possible to extrapolate the current results to those with no legal status or who live in other cities in Türkiye. Second, COVID-19 outbreak happened after the baseline assessments were completed. It might have exacerbated the existing mental health problems (Kurt *et al.*, 2021) though controlled for its impact in the analyses and thereby hindered potential treatment gains. Some of the measures had not been validated in the Syrian population, though they exhibited strong psychometric properties in the present study. This might have limited our ability to capture culture-specific expressions of psychological distress. Furthermore, similar to the bulk of previous research, the predominance of female participants in the trial (69.6% of the sample) necessitates the development of specific strategies to attract more male participants. This is essential to ensure a more generalizable representation of the population.

To conclude, the current findings show the potential utility and limits of gPM+ delivered by non-specialist peer facilitators to alleviate psychological distress among Syrian refugees in Türkiye. This trial is the first to test the effectiveness of brief, scalable, evidence-based group psychosocial intervention for refugees in Türkiye, the major refugee-hosting country with the highest number of Syrians.

## Supporting information

Acarturk et al. supplementary material Acarturk et al. supplementary material

## Data Availability

Data are available on reasonable request. The Vrije Universiteit Amsterdam (VU) will keep a central data repository of all data collected in the STRENGTHS project. The data will be available on reasonable request to the STRENGTHS consortium. Data access might not be granted to third parties when this would interfere with relevant data protection and legislation in the countries participating in this project and any applicable European Union legislation regarding data protection. The PM+ training manual and intervention manual are available through the consortium. Interested researchers can contact prof. Dr. Marit Sijbrandij at e.m.sijbrandij@vu.nl to initiate the process.

## Supplementary material

The supplementary material for this article can be found at https://doi.org/10.1017/S2045796024000453.
