# Supplementary material for: Effectiveness of group problem management plus in distressed Syrian refugees in Türkiye: a randomized controlled trial
Source: Epidemiol Psychiatr Sci. 2024 Sep 30;33:e43. doi: 10.1017/S2045796024000453 (PMC11464952; doi:10.1017/S2045796024000453)
Supplement: Acarturk et al. supplementary material [file S2045796024000453sup001.docx]

**Supplementary Materials**

**S1.** CONSORT 2010 checklist of information to include when reporting a randomized trial.

| **Section/Topic** | **Item No** | **Checklist item** | **Reported on page No** |
| --- | --- | --- | --- |
| **Title and abstract** | | | |
|  | 1a | Identification as a randomised trial in the title | Title |
|  | 1b | Structured summary of trial design, methods, results, and conclusions (for specific guidance see CONSORT for abstracts) | Abstract |
| **Introduction** | | | |
| Background and objectives | 2a | Scientific background and explanation of rationale | Intro: Para 1-3 |
|  | 2b | Specific objectives or hypotheses | Intro: Para 4 |
| **Methods8** | | | |
| Trial design | 3a | Description of trial design (such as parallel, factorial) including allocation ratio | Study Design |
|  | 3b | Important changes to methods after trial commencement (such as eligibility criteria), with reasons | NA |
| Participants | 4a | Eligibility criteria for participants | Participants |
|  | 4b | Settings and locations where the data were collected | Participants |
| Interventions | 5 | The interventions for each group with sufficient details to allow replication, including how and when they were actually administered | Procedure: Para 1-4 |
| Outcomes | 6a | Completely defined pre-specified primary and secondary outcome measures, including how and when they were assessed | Methods |
|  | 6b | Any changes to trial outcomes after the trial commenced, with reasons | NA |
| Sample size | 7a | How sample size was determined | Statistical Analysis: Para 1 |
|  | 7b | When applicable, explanation of any interim analyses and stopping guidelines | NA |
| Randomisation: |  |  |  |
| Sequence generation | 8a | Method used to generate the random allocation sequence | Randomization and blinding |
|  | 8b | Type of randomisation; details of any restriction (such as blocking and block size) | Randomization and blinding |
| Allocation concealment mechanism | 9 | Mechanism used to implement the random allocation sequence (such as sequentially numbered containers), describing any steps taken to conceal the sequence until interventions were assigned | Randomization and blinding |
| Implementation | 10 | Who generated the random allocation sequence, who enrolled participants, and who assigned participants to interventions | Randomization and blinding |
| Blinding | 11a | If done, who was blinded after assignment to interventions (for example, participants, care providers, those assessing outcomes) and how | Randomization and blinding |
|  | 11b | If relevant, description of the similarity of interventions | NA |
| Statistical methods | 12a | Statistical methods used to compare groups for primary and secondary outcomes | Statistical analysis: Para 2-3 |
|  | 12b | Methods for additional analyses, such as subgroup analyses and adjusted analyses | Statistical analysis: Para 4-5 |
| **Results** | | | |
| Participant flow (a diagram is strongly recommended) | 13a | For each group, the numbers of participants who were randomly assigned, received intended treatment, and were analysed for the primary outcome | Results: Para 1, Fig 1 |
|  | 13b | For each group, losses and exclusions after randomisation, together with reasons | Results: Para 1, Fig 1 |
| Recruitment | 14a | Dates defining the periods of recruitment and follow-up | Results: Para 1 |
|  | 14b | Why the trial ended or was stopped | NA |
| Baseline data | 15 | A table showing baseline demographic and clinical characteristics for each group | Table 1 |
| Numbers analysed | 16 | For each group, number of participants (denominator) included in each analysis and whether the analysis was by original assigned groups | Fig 1 |
| Outcomes and estimation | 17a | For each primary and secondary outcome, results for each group, and the estimated effect size and its precision (such as 95% confidence interval) | Results: Para 1-2, Table 3 |
|  | 17b | For binary outcomes, presentation of both absolute and relative effect sizes is recommended | NA |
| Ancillary analyses | 18 | Results of any other analyses performed, including subgroup analyses and adjusted analyses, distinguishing pre-specified from exploratory | Results: Para 3-4, Table 4-5 |
| Harms | 19 | All important harms or unintended effects in each group (for specific guidance see CONSORT for harms) | Results: Para 1 |
| **Discussion** | | | |
| Limitations | 20 | Trial limitations, addressing sources of potential bias, imprecision, and, if relevant, multiplicity of analyses | Disc: Para 7 |
| Generalisability | 21 | Generalisability (external validity, applicability) of the trial findings | Disc: Para 7 |
| Interpretation | 22 | Interpretation consistent with results, balancing benefits and harms, and considering other relevant evidence | Disc: Para 2-3 |
| **Other information** | | |  |
| Registration | 23 | Registration number and name of trial registry | Study Design |
| Protocol | 24 | Where the full trial protocol can be accessed, if available | Ref #3 |
| Funding | 25 | Sources of funding and other support (such as supply of drugs), role of funders | Source of funding |

**S2.** Percentages of Experiencing Potentially Traumatic Experiences among Participants

| Items | N (%) |
| --- | --- |
| 1. Natural disaster (for example tornado, hurricane, flood or major earthquake) | 87 (23.6) |
| 2. Serious accident, fire, or explosion (for example, industrial, farm, car, plane or boating accident) | 165 (44.8) |
| 3. Life-threatening illness | 74 (20.1) |
| 4. Serious physical injury | 70 (19) |
| 5. Having fought in a combat situation | 23 (6.3) |
| 6. Being a civilian in a war zone | 260 (70.7) |
| 7. Imprisonment (for example, prison inmate, prisoner of war, hostage) | 32 (8.7) |
| 8. Having been kidnapped | 14 (3.8) |
| 9. Having been in a refugee camp | 41 (11.1) |
| 10. Having been in danger during the flight (see, boat, border) | 201 (54.6) |
| 11. Non-sexual assault by a family member or someone you know (for example, being mugged, physically attacked, shot, stabbed, or held at gunpoint) | 17 (4.6) |
| 12. Non-sexual assault by a stranger (for example being mugged, physically attacked, shot, stabbed, or held at gunpoint) | 31 (8.4) |
| 13. Enforced isolation from others | 36 (9.8) |
| 14. Brainwashing | 10 (2.7) |
| 15. Torture | 31 (8.4) |
| 16. Sexual assault (rape, attempted rape, made to perform any type of sexual act through force or threat of harm) | 10 (2.7) |
| 17. Other unwanted or uncomfortable sexual experience | 10 (2.7) |
| 18. Exposure to toxic substances (for example, dangerous chemicals, radiation) | 24 (6.5) |
| 19. Forced separation from a family member | 131 (35.6) |
| 20. Lack of food or water | 196 (53.3) |
| 21. Lack of shelter | 164 (44.6) |
| 22. Ill health without access to medical care | 103 (28) |
| 23. Murder of a family member or friend | 116 (31.5) |
| 24. Unnatural death of a family member or friend | 150 (40.8) |
| 25. Witnessing the murder of one or more strangers | 85 (23.1) |
| 26. Disappearance of family or friend | 128 (34.8) |
| 27. Any other very stressful event or experience where your life was in danger (not mentioned previously) | 56 (15.2) |

**S3.** Percentages of Experiencing Moderately Serious Post-Displacement Stressors at 3-month follow-up.

| Items | N (%) |
| --- | --- |
| 1. Communication difficulties | 170 (46.2) |
| 2. Discrimination | 170 (46.2) |
| 3. Conflicts with your own/other ethnic groups in [Türkiye] | 32 (8.7) |
| 4. Separation from family | 126 (34.2) |
| 5. Worries about family back at home | 163 (44.3) |
| 6. Unable to return home in an emergency | 141 (38.3) |
| 7. Difficulties with employment (being permitted to work, finding work, bad working conditions, etc.) | 90 (24.5) |
| 8. Difficulties in interviews with immigration officials | 41 (11.1) |
| 9. Conflicts with social workers/other authorities | 25 (6.8) |
| 10. Not being recognized as a refugee | 44 (12) |
| 11. Being fearful of being sent back to your country of origin in the future | 158 (42.9) |
| 12. Worries about not getting access to treatment for health problems | 156 (42.4) |
| 13. Not enough money to buy food, pay the rent, or buy necessary clothes | 262 (71.2) |
| 14. Difficulties obtaining financial assistance | 252 (68.5) |
| 15. Loneliness, boredom, or isolation | 180 (48.9) |
| 16. Difficulties in learning the local [Turkish] language | 228 (62) |
| 17. Difficulties obtaining appropriate accommodation | 154 (41.8) |

**S4.** Results from Mixed-Model Analysis of Primary and Secondary Outcomes with 3-months Completers Only

|  |  | **Descriptive Statistics** | | **Mixed-Model Analysis** | | |
| --- | --- | --- | --- | --- | --- | --- |
|  |  | **Mean (SD)** | **Mean (SD)** |  |  |  |
| **Outcome variable** | **Time point** | **gPM+/ECAU (n=153)** | **ECAU**  **(n=156)** | **Difference in mean** | **P value** | **Cohen’s effect size** |
| **HSCL-25 Total** | Baseline | 2.11 (.65) | 2.17 (.60) |  |  |  |
|  | Post | 1.91 (.57) | 2.02 (.63) | -0.08 (-0.20, 0.04) | 0.201 | 0.13 |
|  | 3-month | 2.08 (.59) | 2.13 (.62) | -0.02 (-0.14, 0.09) | 0.711 | 0.03 |
| **HSCL-25 Depression** | Baseline | 2.10 (.67) | 2.15 (.62) |  |  |  |
|  | Post | 1.89 (.59) | 2.00 (.66) | -0.09 (-0.22, 0.04) | 0.177 | 0.14 |
|  | 3-month | 2.07 (.62) | 2.11 (.66) | -0.01 (-0.14, 0.11) | 0.846 | 0.02 |
| **HSCL-25 Anxiety** | Baseline | 2.12 (.72) | 2.20 (.69) |  |  |  |
|  | Post | 1.95 (.64) | 2.05 (.71) | -0.07 (-0.20, 0.07) | 0.344 | 0.10 |
|  | 3-month | 2.09 (.64) | 2.17 (.67) | -0.04 (-0.17, 0.09) | 0.572 | 0.04 |
| **PCL-5** | Baseline | 25.76 (17.64) | 28.76 (17.49) |  |  |  |
|  | Post | 22.93 (16.24) | 23.37 (16.63) | 1.05 (-2.41, 4.52) | 0.551 | 0.06 |
|  | 3-month | 24.95 (15.87) | 27.77 (17.86) | -1.30 (-4.63, 2.03) | 0.444 | 0.08 |
| **WHODAS 2.0** | Baseline | 26.81 (7.25) | 26.20 (6.86) |  |  |  |
|  | Post | 23.01 (6.97) | 23.49 (7.79) | -0.90 (-2.48, 0.69) | 0.267 | 0.12 |
|  | 3-month | 23.34 (7.09) | 24.75 (8.02) | -1.64 (-3.16, -0.12) | 0.034* | 0.22 |
| **PSYCHLOPS** | Baseline | 13.96 (5.45) | 15.02 (5.08) |  |  |  |
|  | Post | 12.32 (5.57) | 12.94 (5.68) | -0.33 (-1.65, 0.99) | 0.622 | 0.06 |
|  | 3-month | 11.88 (6.13) | 12.56 (6.46) | -0.47 (-1.74, 0.80) | 0.466 | 0.07 |

**S5.** Results from Mixed-Model Analysis of Primary and Secondary Outcomes with per protocol sample Only

|  |  | **Descriptive Statistics** | | **Mixed-Model Analysis** | | |
| --- | --- | --- | --- | --- | --- | --- |
|  |  | **Mean (SD)** | **Mean (SD)** |  |  |  |
| **Outcome variable** | **Time point** | **gPM+/ECAU (n=135)** | **ECAU**  **(n=184)** | **Difference in mean** | **P value** | **Effect size** |
| **HSCL-25 Total** | Baseline | 2.16 (.62) | 2.16 (.61) |  |  |  |
|  | Post | 1.90 (.55) | 2.02 (.64) | -0.11 (-0.23, 0.01) | 0.073 | 0.18 |
|  | 3-month | 2.10 (.59) | 2.13 (.62) | -0.02 (-0.15, 0.10) | 0.673 | 0.03 |
| **HSCL-25 Depression** | Baseline | 2.14 (.63) | 2.15 (.64) |  |  |  |
|  | Post | 1.87 (.58) | 1.99 (.65) | -0.10 (-0.23, 0.02) | 0.115 | 0.16 |
|  | 3-month | 2.09 (.62) | 2.11 (.66) | -0.01 (-0.14, 0.12) | 0.839 | 0.02 |
| **HSCL-25 Anxiety** | Baseline | 2.19 (.71) | 2.18 (.70) |  |  |  |
|  | Post | 1.94 (.61) | 2.06 (.71) | -0.12 (-0.25, 0.02) | 0.095 | 0.18 |
|  | 3-month | 2.12 (.65) | 2.17 (.67) | -0.04 (-0.18, 0.10) | 0.564 | 0.06 |
| **PCL-5** | Baseline | 27.64 (17.92) | 28.76 (17.76) |  |  |  |
|  | Post | 23.54 (16.16) | 22.81 (16.84) | 1.42 (-2.05, 4.89) | 0.423 | 0.09 |
|  | 3-month | 26.62 (16.19) | 27.77 (17.86) | -0.21 (-3.79, 3.38) | 0.911 | 0.01 |
| **WHODAS 2.0** | Baseline | 27.15 (7.07) | 25.77 (6.67) |  |  |  |
|  | Post | 22.65 (6.67) | 23.31 (7.85) | -1.25 (-2.74, -0.38) | 0.117 | 0.18 |
|  | 3-month | 23.94 (7.12) | 24.75 (8.02) | -1.17 (-2.82, 0.31) | 0.158 | 0.15 |
| **PSYCHLOPS** | Baseline | 14.05 (5.49) | 14.70 (5.44) |  |  |  |
|  | Post | 12.10 (5.18) | 12.61 (5.86) | -0.33 (-1.64, 0.99) | 0.625 | 0.06 |
|  | 3-month | 11.80 (6.24) | 12.56 (6.46) | -0.52 (-1.90, 0.85) | 0.455 | 0.08 |

**S6.** Results from Mixed-Model Analysis of Primary and Secondary Outcomes for Participants with Probable Depression or Anxiety

|  |  | **Descriptive Statistics** | | **Mixed-Model Analysis** | | |
| --- | --- | --- | --- | --- | --- | --- |
|  |  | **Mean (SD)** | **Mean (SD)** |  |  |  |
| **Outcome variable** | **Time point** | **gPM+/ECAU**  **(n=114)** | **ECAU**  **(n=119)** | **Difference in mean** | **P value** | **Effect size** |
| **HSCL-25 Total** | Baseline | 2.50 (.49) | 2.50 (.45) |  |  |  |
|  | Post | 2.03 (.60) | 2.22 (.63) | -0.19 (-0.32, -0.05) | 0.008** | 0.31 |
|  | 3-month | 2.26 (.58) | 2.32 (.58) | -0.07 (-0.21, 0.08) | 0.363 | 0.12 |
| **HSCL-25 Depression** | Baseline | 2.46 (.56) | 2.47 (53) |  |  |  |
|  | Post | 1.99 (.62) | 2.17 (.65) | -0.17 (-0.32, -0.02) | 0.028* | 0.27 |
|  | 3-month | 2.26 (.60) | 2.31 (.63) | -0.04 (-0.20, 0.11) | 0.586 | 0.07 |
| **HSCL-25 Anxiety** | Baseline | 2.55 (.55) | 2.55 (.54) |  |  |  |
|  | Post | 2.09 (.67) | 2.30 (.72) | -0.21 (-0.37, -0.05) | 0.009** | 0.30 |
|  | 3-month | 2.26 (.65) | 2.35 (.63) | -0.10 (-0.26, 0.06) | 0.251 | 0.16 |
| **PCL-5** | Baseline | 35.07 (16.46) | 36.62 (15.33) |  |  |  |
|  | Post | 25.95 (17.92) | 26.18 (17.30) | 0.59 (-3.54, 4.72) | 0.779 | 0.03 |
|  | 3-month | 30.35 (15.72) | 32.30 (17.24) | -1.17 (-5.42, 3.08) | 0.589 | 0.07 |
| **WHO-DAS** | Baseline | 28.35 (7.31) | 27.05 (6.11) |  |  |  |
|  | Post | 24.27 (7.18) | 24.78 (7.99) | -1.04 (-2.94, 0.86) | 0.284 | 0.14 |
|  | 3-month | 24.83 (7.31) | 26.50 (7.88) | -1.98 (-3.93, -0.02) | 0.048* | 0.26 |
| **PSYCHLOPS** | Baseline | 15.25 (4.77) | 15.82 (4.81) |  |  |  |
|  | Post | 13.09 (5.19) | 13.05 (5.97) | 0.17 (-1.30, 1.63) | 0.824 | 0.03 |
|  | 3-month | 12.83 (5.76) | 13.66 (6.11) | -0.69 (-2.20, 0.82) | 0.373 | 0.12 |

| **S7:** Mean Difference in Cumulative Service Utilisation Per Participant at 3-Month Follow-Up | | | | |
| --- | --- | --- | --- | --- |
| Type of Cost | gpm+/E-CAU  (184) | E-CAU (184) | Mean Difference (BCa* 95% CI) | p |
| Health Service Utilisation, M (SD) | | | | |
| Community health worker (contact) | 0.85 (2.44) | 0.79 (1.74) | 0.06 (-0.33, 0.50) | 0.810 |
| Community-based doctor (contact) | 0.63 (1.81) | 0.51 (1.39) | 0.12 (-0.20, 0.45) | 0.496 |
| Psychiatrist (contact) | 0.07 (0.53) | 0.18 (1.08) | -0.10 (-0.32, 0.05) | 0.250 |
| Psychologist (contact) | 0.06 (0.60) | 0.02 (0.15) | 0.04 (-0.21, 0.16) | 0.406 |
| Psychiatric Nurse (contact) | 0.03 (0.15) | 0.00 (0.00) | 0.03 (-0.01, 0.07) | 0.159 |
| Social worker (contact) | 0.04 (0.22) | 0.00 (0.00) | 0.04 (0.00, 0.07) | 0.098 |
| Psychiatric inpatient stay (nights) | 0.05 (0.33) | 0.04 (0.34) | 0.02 (-0.05, 0.08) | 0.637 |
| Other inpatient stay (nights) | 0.16 (0.88) | 0.21 (1.20) | -0.05 (-0.27, 0.16) | 0.621 |
| Hospital Emergency Department (contact) | 0.26 (0.94) | 0.16 (0.67) | 0.09 (-0.08, 0.26) | 0.279 |
| Psychiatric outpatient (contact) | 0.00 (0.00) | 0.00 (0.00) |  |  |
| Other outpatient (contact) | 0.35 (2.13) | 0.24 (0.96) | 0.11 (-0.23, 0.45) | 0.518 |
| Day Hospital (Visit) | 0.31 (1.66) | 0.33 (1.66) | -0.02 (-0.34, 0.30) | 0.931 |
| Policlinic (Visit) | 0.33 (1.15) | 0.30 (1.22) | 0.03 (-0.23, 0.29) | 0.802 |
| CAM (contact) | 0.36 (0.93) | 0.65 (5.58) | -0.29 (-1.50, 0.31) | 0.560 |
| Productivity Loss (days) | 4.41 (15.26) | 2.65 (10.47) | 1.76 (-0.53, 4.08) | 0.198 |
